# Biomechanical evaluation on a new type of vertebral titanium porous mini-plate and mechanical comparison between cervical open-door laminoplasty and laminectomy: a finite element analysis

**DOI:** 10.3389/fbioe.2024.1353797

**Published:** 2024-02-05

**Authors:** Zhiwei Lin, Dongxin Lin, Lin Xu, Qiwei Chen, Manoj Kumar Vashisth, Xuecheng Huang, Yuping Deng, Feihu Zhang, Wenhua Huang

**Affiliations:** ^1^ School of Basic Medical Sciences, Guangdong Medical University, Dongguan, China; ^2^ Guangdong Provincial Key Laboratory of Digital Medicine and Biomechanics, National Key Discipline of Human Anatomy, Guangdong Engineering Research Center for Translation of Medical 3D Printing Application, School of Basic Medical Sciences, Southern Medical University, Guangzhou, China; ^3^ Department of Orthopaedic, The First Hospital of Qiqihar, Heilongjiang, China; ^4^ Shenzhen Hospital of Guangzhou University of Chinese Medicine (Futian), Shenzhen, China; ^5^ Integrated Hospital of Traditional Chinese Medicine, Southern Medical University, Guangzhou, China

**Keywords:** finite element analysis, biomechanics, laminectomy, laminoplasty, titanium miniplate, cervical spondylotic myelopathy

## Abstract

**Objective:** Compare the spine’s stability after laminectomy (LN) and laminoplasty (LP) for two posterior surgeries. Simultaneously, design a new vertebral titanium porous mini plate (TPMP) to achieve firm fixation of the open-door vertebral LP fully. The objective is to enhance the fixation stability, effectively prevent the possibility of “re-closure,” and may facilitate bone healing.

**Methods:** TPMP was designed by incorporating a fusion body and porous structures, and a three-dimensional finite element cervical model of C2-T1 was constructed and validated. Load LN and LP finite element models, respectively, and analyze and simulate the detailed processes of the two surgeries. It was simultaneously implanting the TPMP into LP to evaluate its biomechanical properties.

**Results:** We find that the range of motion (ROM) of C4-C5 after LN surgery was greater than that of LP implanted with different plates alone. Furthermore, flexion-extension, lateral bending, and axial rotation reflect this change. More noteworthy is that LN has a much larger ROM on C2-C3 in axial rotation. The ROM of LP implanted with two different plates is similar. There is almost no difference in facet joint stress in lateral bending. The facet joint stress of LN is smaller on C2-C3 and C4-C5, and larger more prominent on C5-C6 in the flexion-extension. Regarding intervertebral disc pressure (IDP), there is little difference between different surgeries except for the LN on C2-C3 in axial rotation. The plate displacement specificity does not significantly differ from LP with vertebral titanium mini-plate (TMP) and LP with TPMP after surgery. The stress of LP with TPMP is larger in C4-C5, C5-C6. Moreover, LP with TMP shows greater stress in the C3-C4 during flexion-extension and lateral bending.

**Conclusion:** LP may have better postoperative stability when posterior approach surgery is used to treat CSM; at the same time, the new type of vertebral titanium mini-plate can achieve almost the same effect as the traditional titanium mini-plate after surgery for LP. In addition, it has specific potential due to the porous structure promoting bone fusion.

## 1 Introduction

Cervical Spondylotic Myelopathy (CSM) is a type of cervical spondylosis, which mainly refers to the degeneration of the intervertebral connection structure of the cervical spine, resulting in spinal cord compression or ischemia and, subsequently, spinal cord dysfunction (Xiong et al., 2015; Ghogawala et al., 2021). It is critical to realize that cervical spinal cord injury could be traumatic or non-traumatic. Cervical spondylosis with neuropathy and myelopathy comes under non traumatic spinal cord injury (Goel et al., 2018; Tyagi et al., 2019). CSM accounts for 10%–15% of cervical spondylosis and is the most common cause of spinal cord dysfunction worldwide (Badhiwala et al., 2020). Currently, the main posterior surgical methods for CSM include LN and LP. The purpose of LP is to open and expand the vertebral canal, causing the cervical spinal cord to drift backwards and alleviating the patient’s symptoms (Wang et al., 2022). Lumbar LN and fusion can expand the spinal canal, shift the cervical segment of the spinal cord backwards, release pressure, and significantly stabilize the cervical spine. The computed endpoints may not be adequate to make firm conclusions, although several prior meta-analyses have compared LP and LF in the treatment of CSM and ossification of the posterior longitudinal ligament (Lee et al., 2016; Ma et al., 2018; Yuan et al., 2019). There is currently limited research on the biomechanical effects of the cervical spine after LN and LP. In the original LP outlined by Hirabayashi, the vertebral lamina is reconstructed through suturing and fixation. Although the long-term neurological results of cervical LP with suture fixation have been satisfactory (Suk et al., 2007; Okada et al., 2009), LP re-closure is also considered a problem related to this surgery. Matsumoto et al. (Matsumoto et al., 2008) reported that up to 34% of patients have varying degrees of lamina re-closure at one or more segments after LP using suture fixation. There are also some clinical reports indicating the risk of re-closure between the vertebral lamina and lateral mass (Wang et al., 2011). At the same time, existing LP also has some defects, such as the risk of re-closure of the vertebra, the possibility of intraoperative self-bone transplantation, and the inability of the solid structure of the spacer to promote bone fusion.

Cervical biomechanics research primarily uses *in vitro* and *in vivo* models. Obtaining human specimens is exceedingly tricky because of medical ethics and conventional ethics limits, even though body specimens have good human representativeness and can effectively support cervical biomechanics research (Cho et al., 2022; Silva et al., 2023). Furthermore, the broad restrictions imposed by medical ethics restrict the utilization of human living models. However, the progression of science and technology has facilitated the introduction of computer simulation technology and finite element analysis methods, presenting novel approaches and technologies for investigating cervical biomechanics. (Sun et al., 2022; Gerringer et al., 2023). Finite element analysis can be utilized to compare the biomechanical properties of the cervical spine under physiological or pathological conditions by altering parameters and analyzing their effects. This enables an examination of pathological processes’ impact on the cervical spine’s mechanical characteristics (Srinivasan et al., 2021; Frantsuzov et al., 2023; Hsieh et al., 2023). This method can evaluate the biomechanical effects of various spinal surgeries and assess the mechanical stability of different implants by calculating and analyzing parameters such as ROM, IDP, facet joint stress, and stress in the spinal cord, among other factors. (Chen et al., 2020; Lin et al., 2023; Wang et al., 2023).In this study, we developed finite element models of the healthy C2-T1, C3-C6 LN, C3-C6 LP, and C3-C6 LP with vertebral TPMP. This research aims to improve and optimize the current vertebral plate fixation system, devise a novel vertebral plate fixation system, and assess the biomechanical effects following LN and LP.

## 2 Materials and methods

### 2.1 Design process of a new type of vertebral titanium mini-plate

**FIGURE 1:**
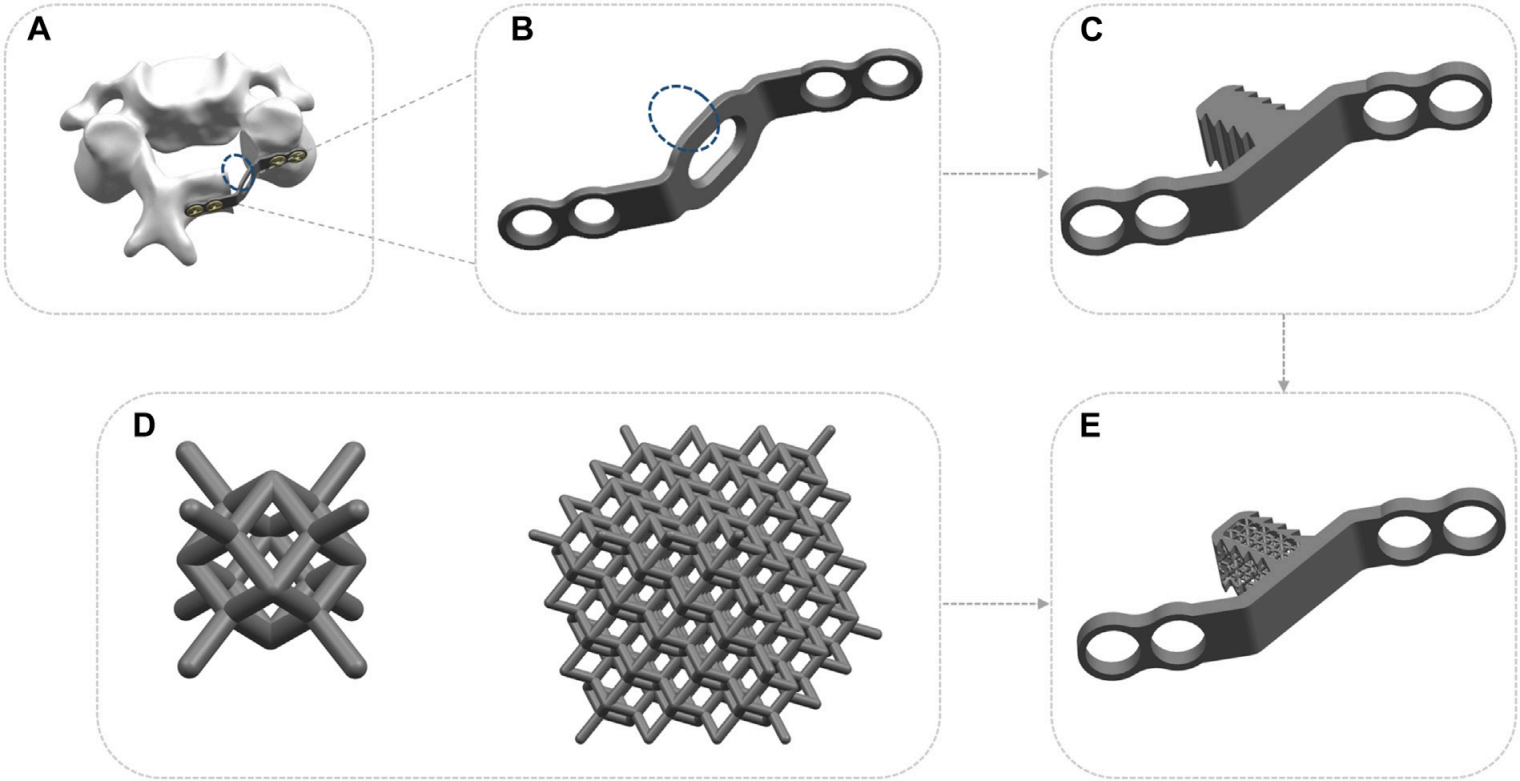
The design process of TPMP. **(A)** Extracting traditional spinal TMP implanted with LP. **(B)** A lack of spacer between the vertebral lamina and lateral mass. **(C)** The combination of fusion body and TMP. **(D)** Fusion body filled with porous structure. **(E)** The overall appearance of TPMP.

The design process is depicted in Figure 1. Initially, the traditional vertebral TMP implanted in LP (Ke et al., 2021; Liang et al., 2023) is extracted (Figure 1A). It has come to our attention that there is a lack of spacer (Figure 1B) between the vertebral lamina and lateral mass. We introduced a fusion body (Figure 1C) to address this issue while incorporating serrations to prevent extraction. Afterwards, fill the fusion body with a porous structure (Figure 1D). The parameters of the porous structure include a small beam diameter of approximately 200 μm. This process completed the design of the vertebral TPMP (Figure 1E).

### 2.2 Establishment of a complete finite element model of the cervical spine

The subject of this study is a healthy volunteer (male, age 26, height 174 cm, weight 66 kg). The participants’ dual-source CT scans were acquired at 0.625 mm intervals (SOMATOM Definition AS +, Siemens, Germany). Furthermore, we established a three-dimensional finite element model of the C2-T1 cervical spine using DICOM data (Ahn et al., 2023). The research was carried out following the guidelines in the Declaration of Helsinki. This study was reviewed by the Medical Ethics Committee of Southern Hospital of Southern Medical University, and the participants signed an informed consent form (license number: 1, date: 2 January 2022).

The complete cervical spine model comprises seven vertebrae, six intervertebral discs, and related ligaments. It is a detailed three-dimensional finite element model based on cross-sectional CT images. The DICOM format imaging files of healthy volunteers should be read by the medical 3D reconstruction software MIMICS 21.0 (Materialize, Leuven, Belgium). Then, reconstruct the geometric structure of the cervical vertebrae through threshold segmentation, editing masks, cavity filling, and other operations. Subsequently, the data was imported into the reverse engineering software Geomagic Studio 2017 (Geomagic, NC, USA) for smoothing, converted into corresponding geometric entities, and exported as an STP file. Then, the C2-T1 vertebral model of the cervical spine was imported into Solidworks 2021 (France, Dassault Company) to generate a computer-aided design (CAD) model of the cervical spine. The models of the cortical bone, cancellous bone, facet joint, fibrous ring, nucleus pulposus, and endplate cartilage were created based on the contours of the cervical spine vertebra (Mo et al., 2015). At last, the finite element model was analyzed using the finite element analysis software Ansys (ANSYS Ltd., Canonsburg, Pennsylvania, United States).

**FIGURE 2:**
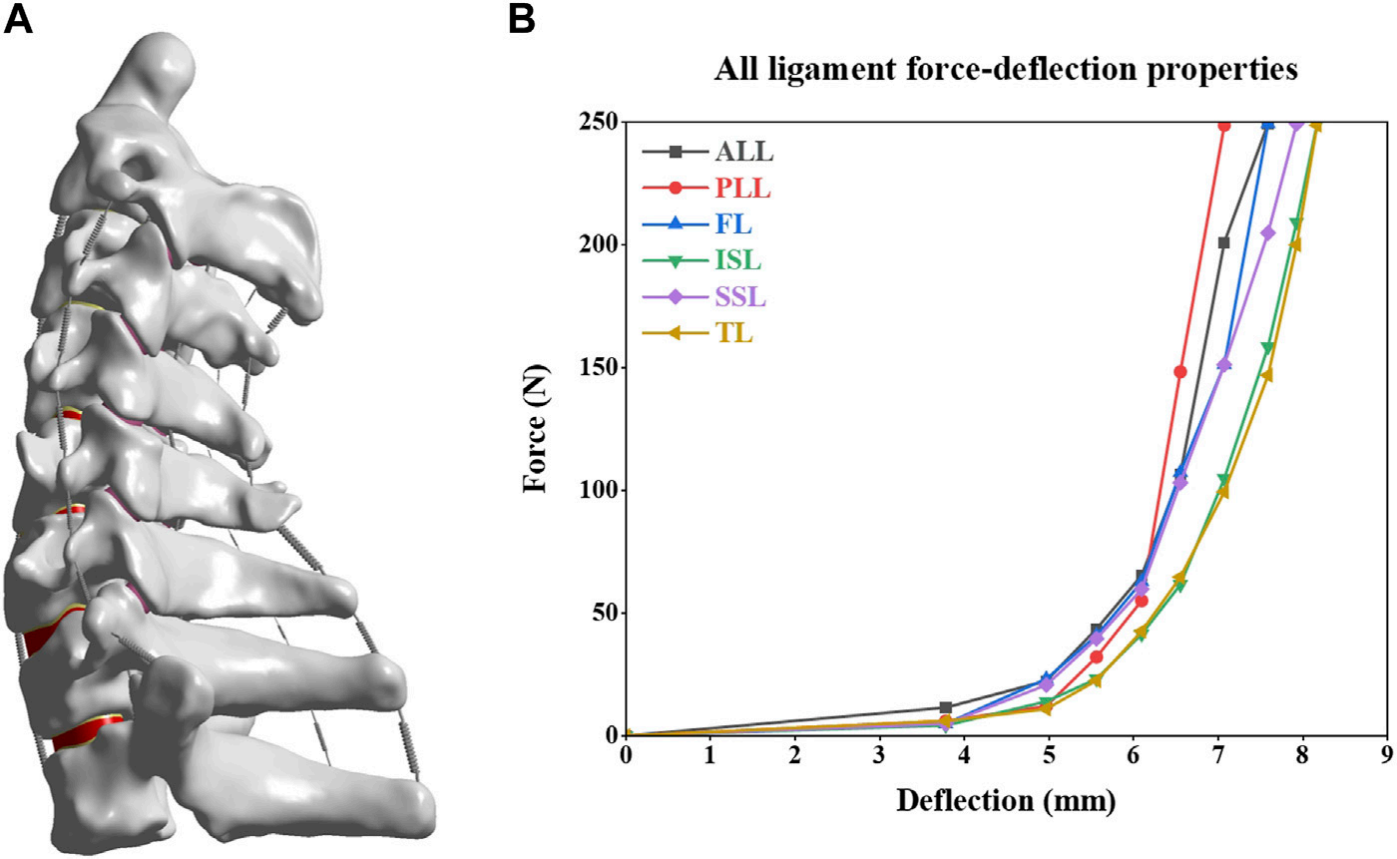
Intact C2-T1 spine finite element model and material properties of the ligaments. **(A)** Intact C2-T1 spine finite element model. **(B)** Material properties of the ligaments.

The C2-T1 finite element model can be divided into cortical bone, cancellous bone, intervertebral disc (IVD), facet joints, and related ligaments (Figure 2A). Cortical bone is constructed as a tetrahedron with a thickness of 0.5 mm (Hua et al., 2020). The intervertebral disc (IVD) comprises annulus fibrosus and nucleus pulposus. The nucleus pulposus is located near the centre of the intervertebral disc and accounts for 40% of the intervertebral disc (Li et al., 2021; Tang et al., 2022). The endplate is a tetrahedron with a thickness of 0.5 mm. The facet joint is recognized as cartilage tissue and has frictionless sliding contact with its upper and lower vertebrae (Lin et al., 2023). The ligaments consist of Anterior longitudinal ligament, Posterior longitudinal ligament, Ligamentum flavum, Interspinous ligament, Supraspinous ligament, and Transverse ligament. These ligaments were established with nonlinear tension only spring elements (Wang et al., 2017; Xu et al., 2022). The material characteristics of the model are listed in Figure 2B and Table 1 (Cai et al., 2020; Guo H. et al., 2021; Guo X. et al., 2021).

**TABLE 1:** Material characteristics of three-dimensional finite element model of cervical spine.

Component	Element type	Young’s modulus (MPa)	Poisson’s ratio
Cortical bone	solid187	12,000	0.29
Cancellous bone	solid187	450	0.29
Facet cartilage	solid187	10.4	0.4
Endplate	solid187	500	0.4
Nucleus pulposus	solid187	1	0.49
Annulus fibrosus	solid187	3.4	0.4
Titanium alloy	solid187	110,000	0.3
Anterior longitudinal Ligament	Spring (tension only)	-	-
Posterior longitudinal Ligament	Spring (tension only)	-	-
Ligamentum flavum	Spring (tension only)	-	-
Interspinous Ligament	Spring (tension only)	-	-
Supraspinous Ligament	Spring (tension only)	-	-
Intertransverse Ligament	Spring (tension only)	-	-

### 2.3 Finite element models of C3-C6 LN

**FIGURE 3:**
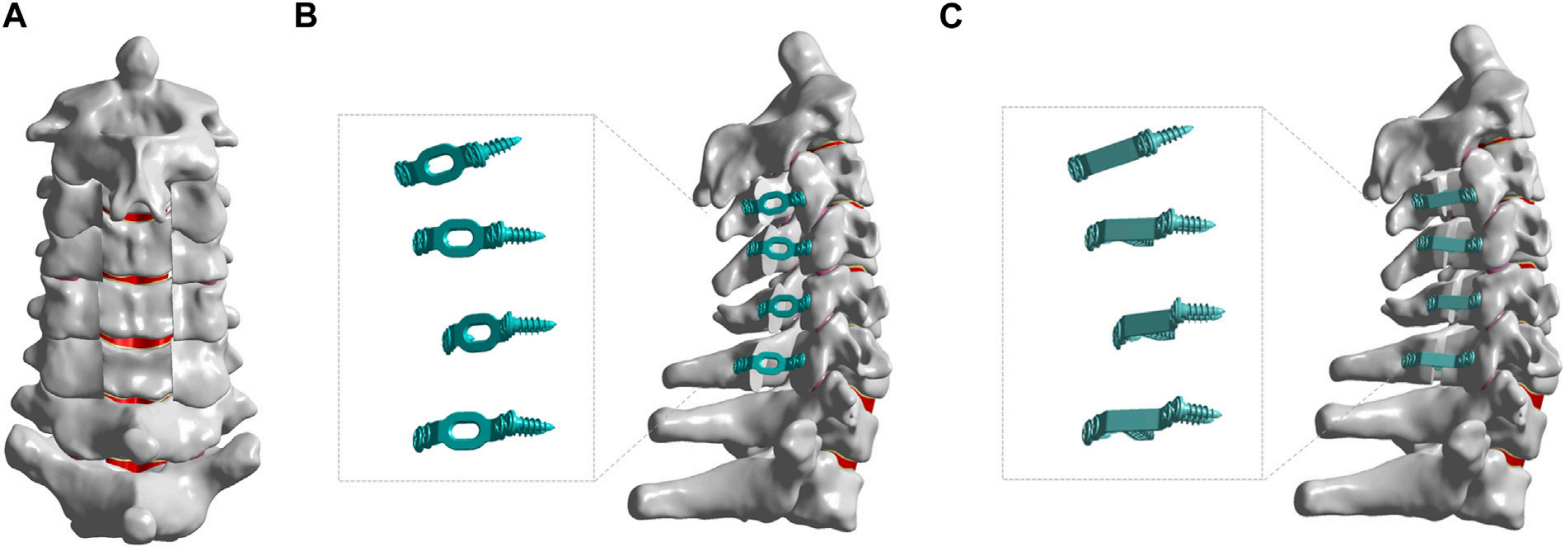
The finite element models of different posterior approach surgeries on C3-C6. **(A)** The finite element models of C3-C6 LN. **(B)** The finite element models of C3-C6 LP + TMP. **(C)** The finite element models of C3-C6 LP + TPMP.

In order to simulate LN on a cervical spine model, a portion of the interspinous ligament (ISL), supraspinal ligament (SSL), and ligamentum flavum (target segment) were removed, and then some of the lamina elements and spinous processes were removed until the medial side of the facet joint was shown (Song et al., 2014; Nishida et al., 2022). This method creates a LN model at the C3–C6 level (Figure 3A).

### 2.4 Finite element models of C3-C6 LP with TMP

As shown in Figure 3B, LP was performed at the C3-C6 segment. This is developed based on the surgical method proposed by Hirabayashi et al. (Hirabayashi et al., 2010). Firstly, remove the ligaments flavum, interspinal ligament, and supraspinal ligament from C3-C6, and then create a V-shaped opening on the hinge side of the vertebral plate, with a width of 12 mm on the opening side. The vertebral plate is fixed with titanium alloy and screws, and the material properties of titanium alloy are as follows: Young’s modulus is 110 Gpa, and Poisson’s ratio is 0.3 (Xu et al., 2022).

### 2.5 Finite element models of C3-C6 LP with TPMP

The surgical method is similar to LP. Replace the traditional TMP with TPMP as an implant, which is fixed with screws and is still considered as titanium alloy material (As shown in Figure 3C).

### 2.6 Boundary and model validation

To validate the intact finite element model of the cervical spine, the lower surface of the T1 vertebra was fixed within 6 degrees of freedom (Mo et al., 2015). Additionally, a vertical load of 73.6 N and a moment of 1.0 Nm were applied on the upper surface of C2 to replicate the spinal movements in forward flexion, backward extension, left and right lateral bending, and axial rotation (Hua et al., 2020; Ke et al., 2021). The range of motion, intervertebral disc pressure, von-Mises stress in the facet joint, and stress in the vertebral plate of the spine were analyzed. Furthermore, the biomechanical effects of various surgical techniques and post-operative implants were investigated.

## 3 Result

### 3.1 Model validation

**FIGURE 4:**
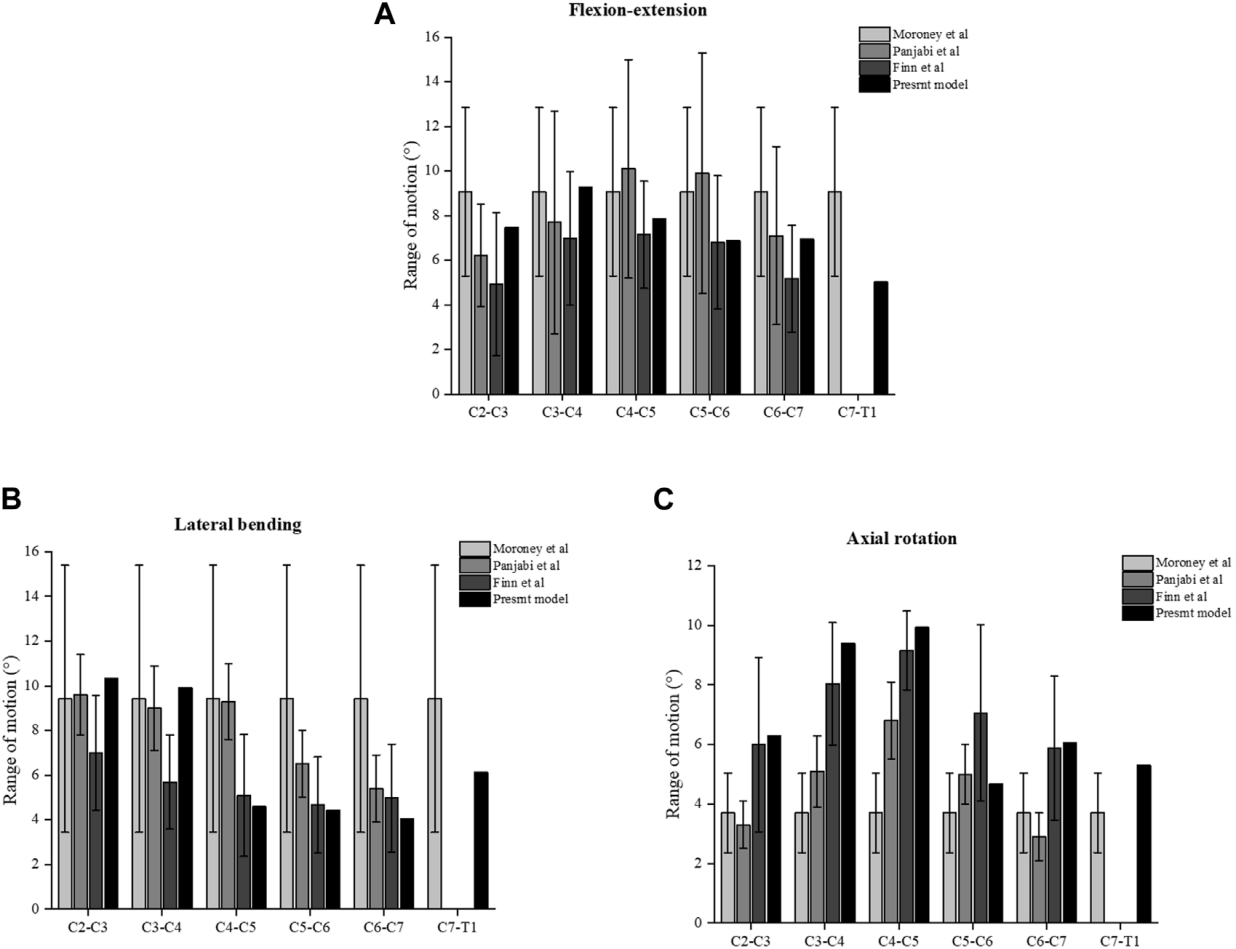
Comparison of the ROM of the intact three-dimensional finite element models of C2-T1 with the prior biomechanical studies. **(A)** ROM in flexion-extension. **(B)** ROM in lateral bending. **(C)** ROM in axial rotation.

Comparative analysis was conducted between the current intact finite element model of the cervical spine and three prior biomechanical studies to assess the efficacy of the aforementioned finite element model (Moroney et al., 1988; Panjabi et al., 2001; Finn et al., 2009). The projected degree of flexion extension, lateral bending, and axial rotation of the entire cervical spine model is congruous with the findings of previous experimental research investigations (Figure 4). A considerably favourable concurrence existed between our experimental data and the reference data. The results indicate that the model can effectively and reasonably predict the biomechanical properties of the cervical spine.

### 3.2 Analysis of biomechanical effects of two different posterior surgical methods for C3-C6 after surgery

**FIGURE 5:**
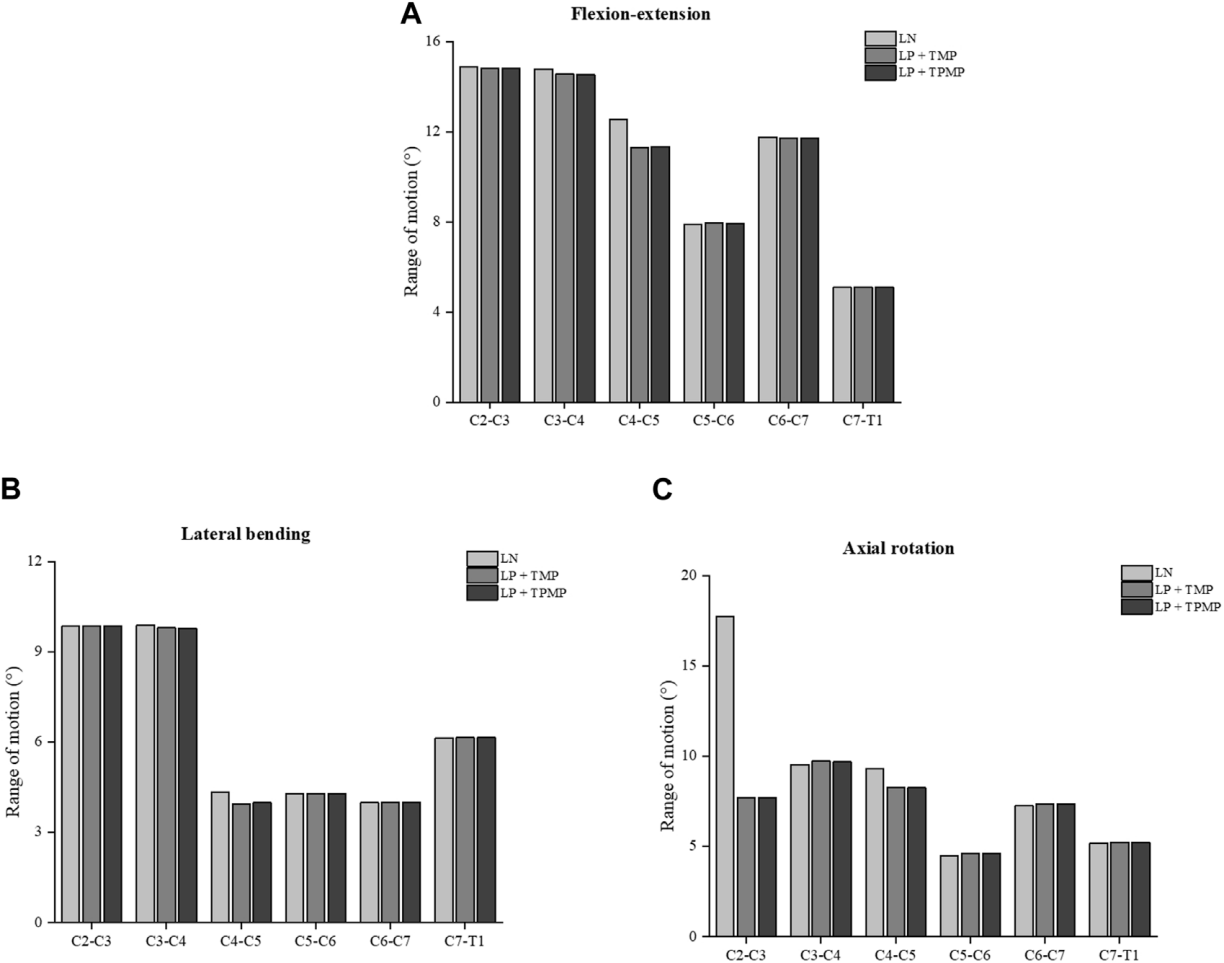
ROM of different posterior approach surgeries on C3-C6. **(A)** ROM in flexion-extension. **(B)** ROM in lateral bending. **(C)** ROM in axial rotation.

**FIGURE 6:**
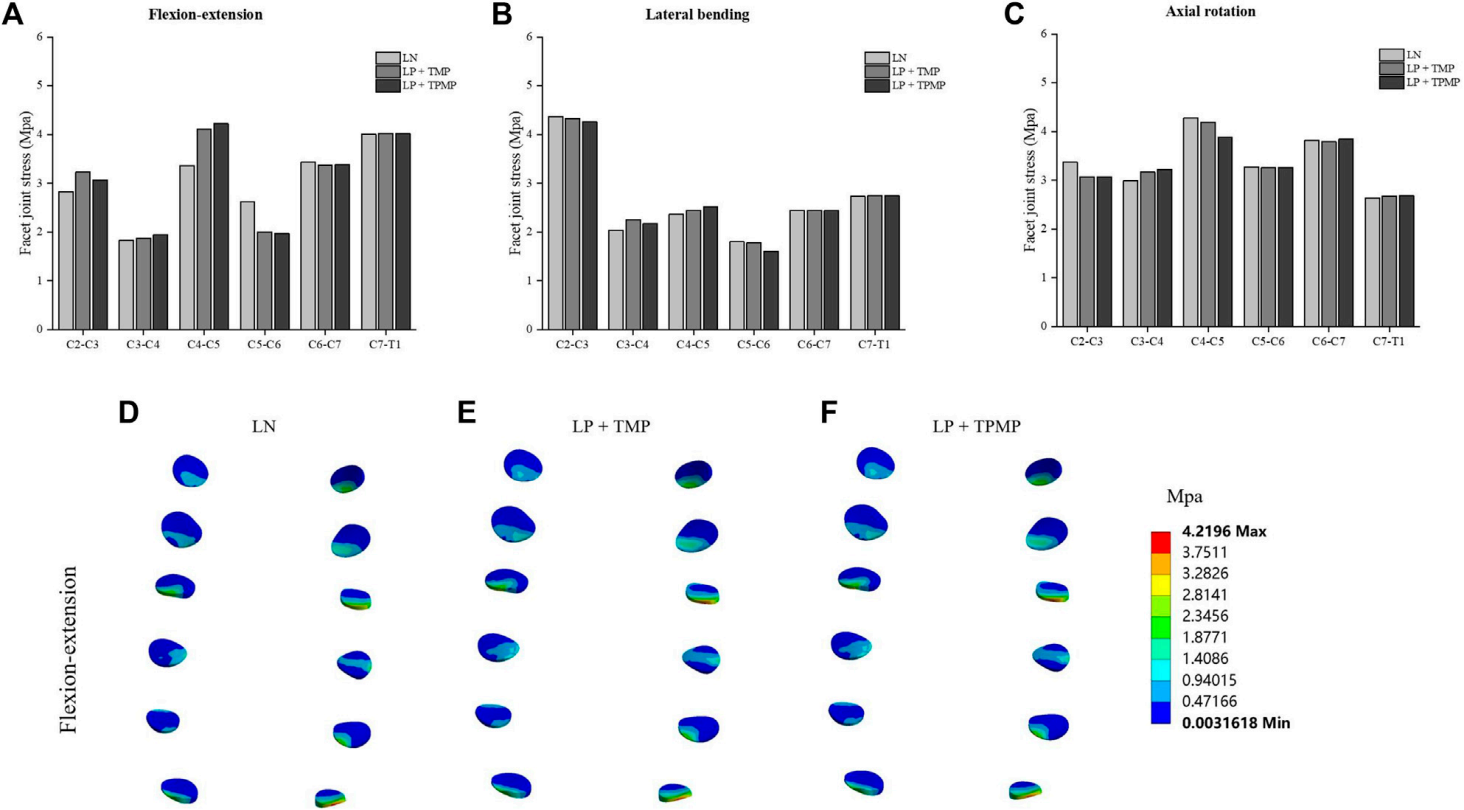
Stress of the facet joints different posterior approach surgeries on C3-C6. **(A)** Stress of the facet joints in flexion-extension. **(B)** Stress of the facet joints in lateral bending. **(C)** Stress of the facet joints in axial rotation. **(D)** Stress distribution of LN in flexion-extension. **(E)** Stress distribution of LP + TMP in flexion-extension. **(F)** Stress distribution of LP + TPMP in flexion-extension.

**FIGURE 7:**
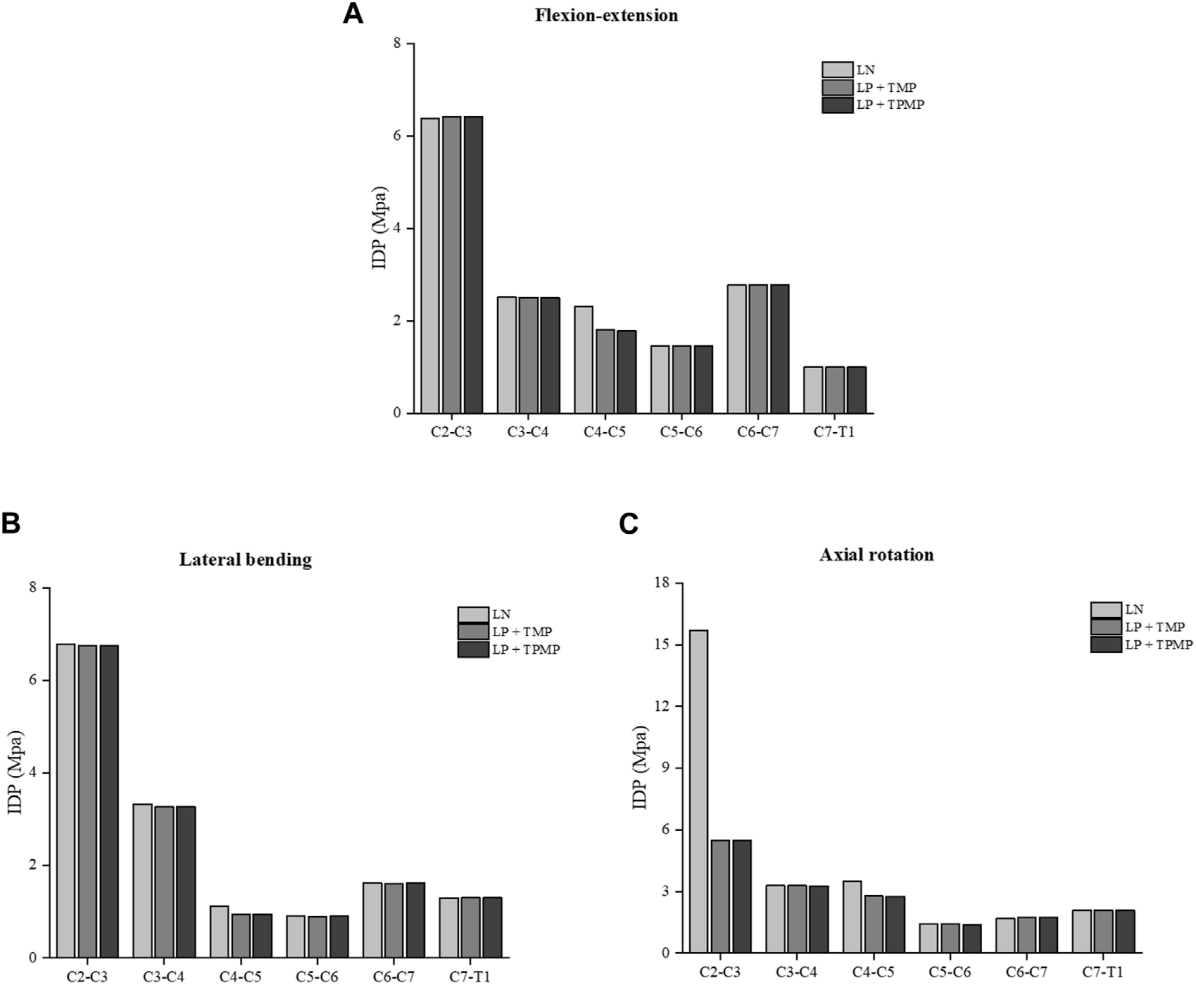
Stress of the IDP of different posterior approach surgeries on C3-C6. **(A)** The IDP in flexion-extension. **(B)** The IDP in lateral bending. **(C)** The IDP in axial rotation.

It can be found that the ROM of C4-C5 after LN surgery surpassed that of LP implanted with different plates alone. This change manifests in flexion-extension, lateral bending, and axial rotation. More noteworthy is that LN has a much larger ROM on C2-C3 in axial rotation. In general, the ROM implanted with two different plates in LP is similar (Figure 5). As for the stress of the facet joints, there is little difference between different surgeries during lateral bending. The facet joint stress of LN is smaller on C2-C3 and C4-C5, and larger more prominent on C5-C6 in the flexion-extension (Figure 6). Regarding intervertebral disc pressure (IDP), there is not much difference between different surgeries except for the LN on C2-C3 in axial rotation (Figure 7).

### 3.3 Biomechanical analysis of C3-C6 LP with TMP and TPMP

**FIGURE 8:**
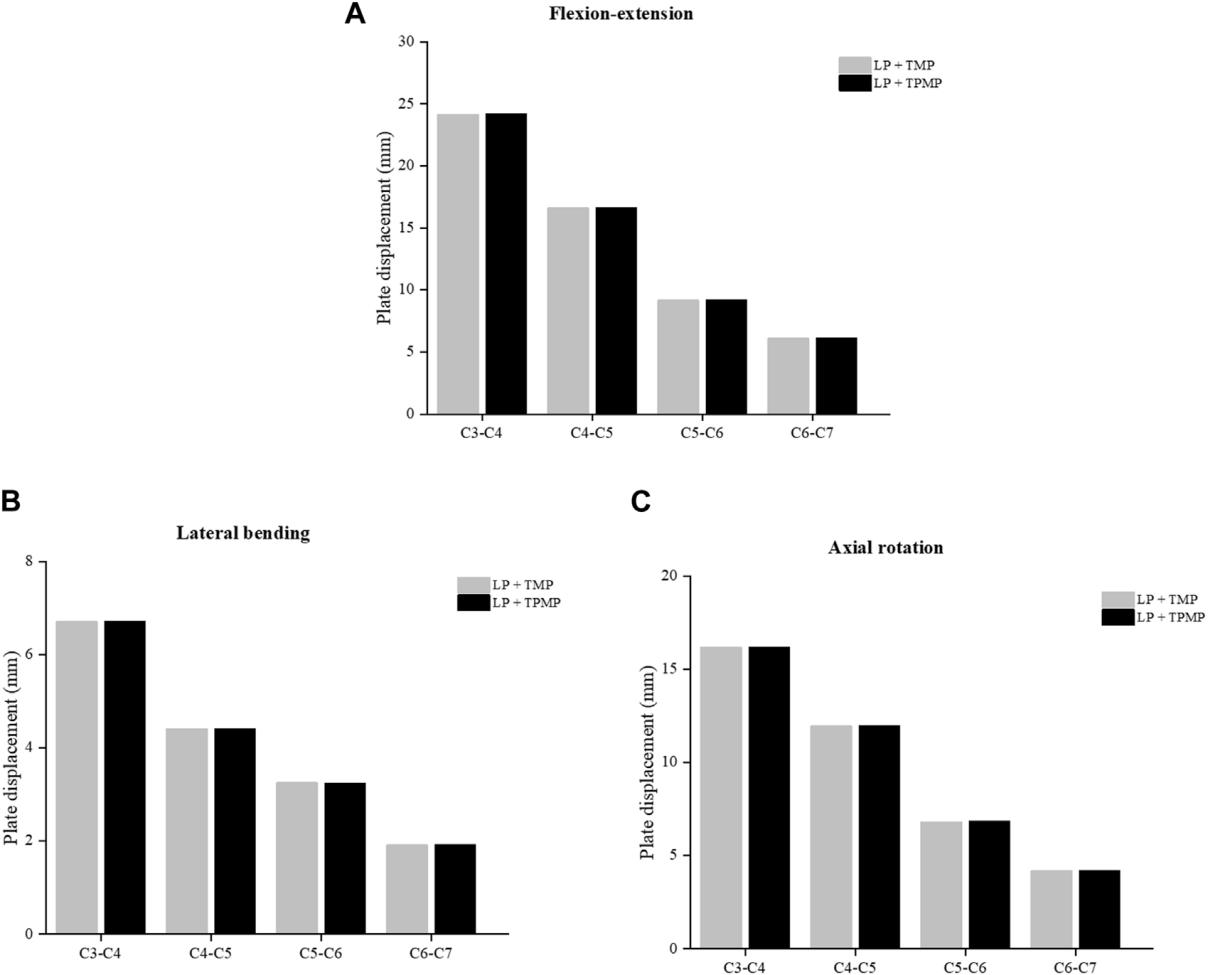
Comparison of the displacement of the C3-C6 LP with TMP and TPMP. **(A)** Displacement in flexion-extension. **(B)** Displacement in lateral bending. **(C)** Displacement in axial rotation.

**FIGURE 9:**
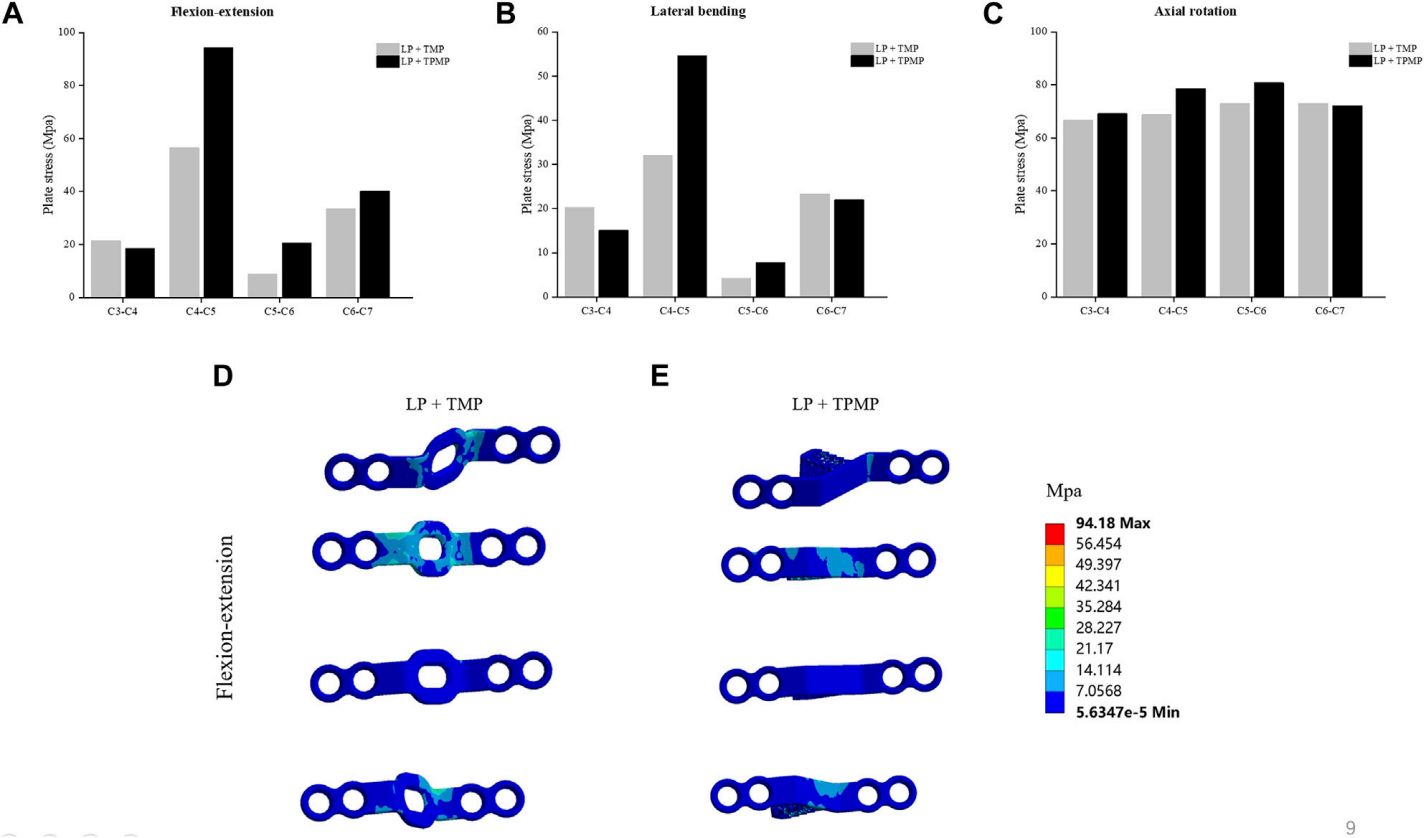
Comparison of the plate von-Mises stress of the C3-C6 LP with TMP and TPMP. **(A)** Plate stress in flexion-extension. **(B)** Plate stress in lateral bending. **(C)** Plate stress in axial rotation. **(D)** Stress distribution of LP + TMP in flexion-extension. **(E)** Stress distribution of LP + TPMP in flexion-extension.

By extracting and comparing data between different implanted plates, it was found that the displacement of the vertebral TMP and TPMP was almost the same, and they both gradually increased with the elevation of the segment (Figure 8). The stress of LP with TPMP is larger in C4-C5, C5-C6. And LP with TMP shows greater stress in the C3-C4 during flexion-extension and lateral bending (Figure 9).

## 4 Discussion

This study aims to compare various biomechanical indicators after LP and LN, as well as the biomechanical effects of LP using TPMP in the treatment of CSM. The biomechanical results indicate that the ROM of C4-C5 after LN surgery was greater than that of LP implanted with different plates alone. Furthermore, flexion-extension, lateral bending, and axial rotation reflect this change. LN results in a significantly more extensive ROM on C2-C3 in axial rotation. The ROM implanted with two different plates in LP is similar. There is almost no difference in facet joint stress in lateral bending. The facet joint stress of LN is smaller on C2-C3 and C4-C5 and more prominent on C5-C6 in the flexion-extension. The facet joint stress of LP with TPMP is smaller on C2-C3 and C4-C5 in the axial rotation compared with LN. This indicates that LP with TPMP can achieve biomechanical effects similar to LN, even better at specific segments and degrees of freedom. Regarding intervertebral disc pressure (IDP), there is little difference between different surgeries except for the LN on C2-C3 in axial rotation. The Increased IDP and facet joint stress of LN on C2-C3 in the axial rotation may be related to ROM. Greater ROM may generate greater stress. TMP and TPMP are almost identical in displacement. The stress of LP with TPMP is larger in C4-C5, C5-C6. And LP with TMP shows greater stress in the C3-C4 during flexion-extension and lateral bending.

The cornerstone of treatment for CSM is spinal canal decompression with cervical LN. This operation aims to expand the back of the cervical spine by removing the spinous processes, lamina, ligamentum flavum, and enlarged bone that cause spinal stenosis (Brown et al., 2021). A dorsal approach is the most effective treatment for patients with congenital spinal stenosis and dorsal compression. In this sense, cervical LN is still a helpful treatment option for CSM (Lu, 2007). Several studies have constructed finite element models for posterior cervical LN and analyzed the resultant alterations in stress distribution within the intervertebral discs and the mobility of the vertebral bodies (Hong-Wan et al., 2004; Ng et al., 2005). Some literature analyses the biomechanical effects and instability based on the range of the posterior bone and ligament complex resection of the LN surgery using finite element technology (Khuyagbaatar et al., 2017). LN was previously considered the “gold standard” for treating CSM, but postoperative cervical instability limited its use (Lu, 2007; Highsmith et al., 2011). In this study, it is possible that LN removed the spinous process and part of the vertebral lamina, resulting in a larger ROM of the entire cervical spine in the flexion and extension direction.

LP is the surgical process of reconstructing the vertebral lamina after opening the spinal canal. The general surgical principle is to create one or more hinges for opening the door, and the vertebral lamina is lifted but not removed on the hinges. This process increases the cross-sectional area of the vertebral canal, relieves spinal cord compression, and then implants multiple segments with vertebral TMPs (Kurokawa and Kim, 2015; Cho et al., 2018). Currently, cervical LN and cervical LP (single or double door) are the main implementation methods of cervical posterior decompression surgery. These two surgical methods have become classic surgeries, but there has yet to be a significant innovation in the specific vertebral TMP for cervical LP in recent decades. In 1997, it was first reported that patients with Hypertrophic spinal pachymeningitis underwent LP, which confirmed that spinal canal decompression and autologous bone graft were acceptable treatment methods for young patients (Kanamori et al., 1997). In recent years, a research team has designed and implemented a technique for inserting an autologous bone spacer between the opened lamina and lateral mass, but without the need for suturing and fixing autologous bone spacer and plates (Kono et al., 2021). Due to the limited number of autologous bone donor sites, long surgical time, and pain in the autologous bone donor site during surgery, HA spacers have been used in LP. This study expands the surgical scope of LP (Goto et al., 2002; Takayasu et al., 2002). In the experimental study of hydroxyapatite/alginate composite injection of three-dimensional polylactic acid scaffolds and mesenchymal stem cells as spacers for LN, the application of the scaffolds has biocompatibility similar to autologous bone graft (Rahyussalim et al., 2022). The above research prompted our team to design a new type of vertebral titanium porous mini-plate, which allows the original TMP to have spacers. Due to the presence of spacers, the risk of vertebral lamina re-closure can be reduced. In addition, TPMP can potentially promote bone fusion due to its porous titanium alloy structure.

There are also some limitations in this study. Firstly, due to the lack of muscles and tendons in this finite element model, it is impossible to simulate various states of the cervical spine accurately. Secondly, the attributes of the intact cervical spine material are outlined as linear and isotropic while ignoring the anisotropy of the material. Therefore, this model has specific differences from the actual human body. Thirdly, a three-dimensional finite element model of a healthy volunteer, which cannot simulate the neck condition of patients with CSM was used as the research object. However, this study can clarify the effects of LN, LP, and LP with TPMP on intervertebral discs and facet joints. Meanwhile, future research should focus on the ability of this TPMP to promote bone fusion. This finite element model helps to infer the application strategies of different posterior cervical surgeries and implants.

## 5 Conclusion

When using posterior surgery to treat CSM, LP may have better immediate postoperative stability than LN. LP can perform spinal cord decompression without significantly altering the cervical ROM. TPMP can achieve biomechanical effects similar to TMP during LP. In addition, due to the presence of porous structures in TPMP that promote bone fusion, it has a particular potential value.

## Data Availability

The original contributions presented in the study are included in the article/Supplementary material, further inquiries can be directed to the corresponding author.

## References

[B1] Ahn C. H. Kang S. Cho M. Kim S. H. Kim C. H. Han I. (2023). Comparing zero-profile and conventional cage and plate in anterior cervical discectomy and fusion using finite-element modeling. Sci. Rep. 13 (1), 15766. 10.1038/s41598-023-43086-x 37737299 PMC10516908

[B2] Badhiwala J. H. Ahuja C. S. Akbar M. A. Witiw C. D. Nassiri F. Furlan J. C. (2020). Degenerative cervical myelopathy - update and future directions. Nat. Rev. Neurol. 16 (2), 108–124. 10.1038/s41582-019-0303-0 31974455

[B3] Brown N. J. Lien B. V. Shahrestani S. Choi E. H. Tran K. Gattas S. (2021). Getting down to the bare bones: does laminoplasty or laminectomy with fusion provide better outcomes for patients with multilevel cervical spondylotic myelopathy? Neurospine 18 (1), 45–54. 10.14245/ns.2040520.260 33819935 PMC8021836

[B4] Cai X. Y. Sang D. Yuchi C. X. Cui W. Zhang C. Du C. F. (2020). Using finite element analysis to determine effects of the motion loading method on facet joint forces after cervical disc degeneration. Comput. Biol. Med. 116, 103519. 10.1016/j.compbiomed.2019.103519 31710870

[B5] Chen C. Yuchi C. X. Gao Z. Ma X. Zhao D. Li J. W. (2020). Comparative analysis of the biomechanics of the adjacent segments after minimally invasive cervical surgeries versus anterior cervical discectomy and fusion: a finite element study. J. Orthop. Transl. 23, 107–112. 10.1016/j.jot.2020.03.006 PMC7322474 32642425

[B6] Cho S. K. Kim J. S. Overley S. C. Merrill R. K. (2018). Cervical laminoplasty: indications, surgical considerations, and clinical outcomes. J. Am. Acad. Orthop. Surg. 26 (7), e142–e152. 10.5435/jaaos-d-16-00242 29521698

[B7] Cho W. Le J. T. Shimer A. L. Werner B. C. Glaser J. A. Shen F. H. (2022). The feasibility of translaminar screws in the subaxial cervical spine: computed tomography and cadaveric validation. Clin. Orthop. Surg. 14 (1), 105–111. 10.4055/cios21059 35251547 PMC8858891

[B8] Finn M. A. Brodke D. S. Daubs M. Patel A. Bachus K. N. (2009). Local and global subaxial cervical spine biomechanics after single-level fusion or cervical arthroplasty. Eur. Spine J. 18 (10), 1520–1527. 10.1007/s00586-009-1085-7 19585159 PMC2899387

[B9] Frantsuzov R. Mondal S. Walsh C. M. Reynolds J. P. Dooley D. MacManus D. B. (2023). A finite element model of contusion spinal cord injury in rodents. J. Mech. Behav. Biomed. Mater 142, 105856. 10.1016/j.jmbbm.2023.105856 37087955

[B10] Gerringer J. W. Somasundaram K. Pintar F. A. (2023). Effect of muscle activation scheme in human head-neck model on estimating cervical spine ligament strain from military volunteer frontal impact data. Accid. Anal. Prev. 190, 107157. 10.1016/j.aap.2023.107157 37336050

[B11] Ghogawala Z. Terrin N. Dunbar M. R. Breeze J. L. Freund K. M. Kanter A. S. (2021). Effect of ventral vs dorsal spinal surgery on patient-reported physical functioning in patients with cervical spondylotic myelopathy: a randomized clinical trial. Jama 325 (10), 942–951. 10.1001/jama.2021.1233 33687463 PMC7944378

[B12] Goel S. A. Modi H. N. Dave B. R. Patel P. R. Patel R. (2018). Socioeconomic impact of cervical spinal cord injury operated in patients with lower income group. Indian Spine J. 1 (1), 46–50. 10.4103/isj.isj_4_17

[B13] Goto T. Ohata K. Takami T. Nishikawa M. Tsuyuguchi N. Morino M. (2002). Hydroxyapatite laminar spacers and titanium miniplates in cervical laminoplasty. J. Neurosurg. 97 (3 Suppl. l), 323–329. 10.3171/spi.2002.97.3.0323 12408386

[B14] Guo H. Li J. Gao Y. Nie S. Quan C. Li J. (2021a). A finite element study on the treatment of thoracolumbar fracture with a new spinal fixation system. Biomed. Res. Int. 2021, 1–9. 10.1155/2021/8872514 33937413 PMC8055395

[B15] Guo X. Zhou J. Tian Y. Kang L. Xue Y. (2021b). Biomechanical effect of different plate-to-disc distance on surgical and adjacent segment in anterior cervical discectomy and fusion - a finite element analysis. BMC Musculoskelet. Disord. 22 (1), 340. 10.1186/s12891-021-04218-4 33836709 PMC8035773

[B16] Highsmith J. M. Dhall S. S. Haid R. W. Jr. Rodts G. E. Jr. Mummaneni P. V. (2011). Treatment of cervical stenotic myelopathy: a cost and outcome comparison of laminoplasty versus laminectomy and lateral mass fusion. J. Neurosurg. Spine 14 (5), 619–625. 10.3171/2011.1.Spine10206 21388285

[B17] Hirabayashi S. Yamada H. Motosuneya T. Watanabe Y. Miura M. Sakai H. (2010). Comparison of enlargement of the spinal canal after cervical laminoplasty: open-door type and double-door type. Eur. Spine J. 19 (10), 1690–1694. 10.1007/s00586-010-1369-y 20309712 PMC2989222

[B18] Hong-Wan N. Ee-Chon T. Qing-Hang Z. (2004). Biomechanical effects of C2-C7 intersegmental stability due to laminectomy with unilateral and bilateral facetectomy. Spine (Phila Pa 1976) 29 (16), 1737–1745. ; discussion 1746. 10.1097/01.brs.0000134574.36487.eb 15303016

[B19] Hsieh M. K. Tai C. L. Li Y. D. Lee D. M. Lin C. Y. Tsai T. T. (2023). Finite element analysis of optimized novel additively manufactured non-articulating prostheses for cervical total disc replacement. Front. Bioeng. Biotechnol. 11, 1182265. 10.3389/fbioe.2023.1182265 37324423 PMC10267663

[B20] Hua W. Zhi J. Ke W. Wang B. Yang S. Li L. (2020). Adjacent segment biomechanical changes after one- or two-level anterior cervical discectomy and fusion using either a zero-profile device or cage plus plate: a finite element analysis. Comput. Biol. Med. 120, 103760. 10.1016/j.compbiomed.2020.103760 32421657

[B21] Kanamori M. Matsui H. Terahata N. Tsuji H. (1997). Hypertrophic spinal pachymeningitis. A case report. Spine (Phila Pa 1976) 22 (15), 1787–1790. 10.1097/00007632-199708010-00021 9259792

[B22] Ke W. Chen C. Wang B. Hua W. Lu S. Song Y. (2021). Biomechanical evaluation of different surgical approaches for the treatment of adjacent segment diseases after primary anterior cervical discectomy and fusion: a finite element analysis. Front. Bioeng. Biotechnol. 9, 718996. 10.3389/fbioe.2021.718996 34532313 PMC8438200

[B23] Khuyagbaatar B. Kim K. Park W. M. Lee S. Kim Y. H. (2017). Increased stress and strain on the spinal cord due to ossification of the posterior longitudinal ligament in the cervical spine under flexion after laminectomy. Proc. Inst. Mech. Eng. H. 231 (9), 898–906. 10.1177/0954411917718222 28660796

[B24] Kono H. Matsuda H. Maeno T. Iwamae M. Nakamura H. (2021). Open-door laminoplasty with stand-alone autologous bone spacers: evaluation of enlarged laminar arch with CT-multiplanar reconstruction. J. Neurosurg. Spine 35 (5), 633–637. 10.3171/2021.1.Spine201633 34359031

[B25] Kurokawa R. Kim P. (2015). Cervical laminoplasty: the history and the future. Neurol. Med. Chir. (Tokyo) 55 (7), 529–539. 10.2176/nmc.ra.2014-0387 26119898 PMC4628185

[B26] Lee C. H. Jahng T. A. Hyun S. J. Kim K. J. Kim H. J. (2016). Expansive laminoplasty versus laminectomy alone versus laminectomy and fusion for cervical ossification of the posterior longitudinal ligament: is there a difference in the clinical outcome and sagittal alignment? Clin. Spine Surg. 29 (1), E9–E15. 10.1097/bsd.0000000000000058 25075990

[B27] Li Z. Liu H. Yang M. Zhang W. (2021). A biomechanical analysis of four anterior cervical techniques to treating multilevel cervical spondylotic myelopathy: a finite element study. BMC Musculoskelet. Disord. 22 (1), 278. 10.1186/s12891-021-04150-7 33722229 PMC7962321

[B28] Liang Z. Xu G. Liu T. Zhong Y. Mo F. Li Z. (2023). Quantitatively biomechanical response analysis of posterior musculature reconstruction in cervical single-door laminoplasty. Comput. Methods Programs Biomed. 233, 107479. 10.1016/j.cmpb.2023.107479 36933316

[B29] Lin D. He Z. Weng R. Zhu Y. Lin Z. Deng Y. (2023). Comparison of biomechanical parameters of two Chinese cervical spine rotation manipulations based on motion capture and finite element analysis. Front. Bioeng. Biotechnol. 11, 1195583. 10.3389/fbioe.2023.1195583 37576989 PMC10415076

[B30] Lu J. J. (2007). Cervical laminectomy: technique. Neurosurgery 60 (1 Suppl. 1 1), S1–S149. 10.1227/01.Neu.0000249219.72956.C7 17204877

[B31] Ma L. Liu F. Y. Huo L. S. Zhao Z. Q. Sun X. Z. Li F. (2018). Comparison of laminoplasty versus laminectomy and fusion in the treatment of multilevel cervical ossification of the posterior longitudinal ligament: a systematic review and meta-analysis. Med. Baltim. 97 (29), e11542. 10.1097/md.0000000000011542 PMC6086468 30024545

[B32] Matsumoto M. Watanabe K. Tsuji T. Ishii K. Takaishi H. Nakamura M. (2008). Risk factors for closure of lamina after open-door laminoplasty. J. Neurosurg. Spine 9 (6), 530–537. 10.3171/spi.2008.4.08176 19035744

[B33] Mo Z. Zhao Y. Du C. Sun Y. Zhang M. Fan Y. (2015). Does location of rotation center in artificial disc affect cervical biomechanics? Spine (Phila Pa 1976) 40 (8), E469–E475. 10.1097/brs.0000000000000818 25868102

[B34] Moroney S. P. Schultz A. B. Miller J. A. Andersson G. B. (1988). Load-displacement properties of lower cervical spine motion segments. J. Biomech. 21 (9), 769–779. 10.1016/0021-9290(88)90285-0 3053721

[B35] Ng H. W. Teo E. C. Zhang Q. (2005). Influence of cervical disc degeneration after posterior surgical techniques in combined flexion-extension--a nonlinear analytical study. J. Biomech. Eng. 127 (1), 186–192. 10.1115/1.1835364 15868801

[B36] Nishida N. Mumtaz M. Tripathi S. Kelkar A. Kumaran Y. Sakai T. (2022). Biomechanical analysis of laminectomy, laminoplasty, posterior decompression with instrumented fusion, and anterior decompression with fusion for the kyphotic cervical spine. Int. J. Comput. Assist. Radiol. Surg. 17 (9), 1531–1541. 10.1007/s11548-022-02692-2 35723866

[B37] Okada M. Minamide A. Endo T. Yoshida M. Kawakami M. Ando M. (2009). A prospective randomized study of clinical outcomes in patients with cervical compressive myelopathy treated with open-door or French-door laminoplasty. Spine (Phila Pa 1976) 34 (11), 1119–1126. 10.1097/BRS.0b013e31819c3b61 19444058

[B38] Panjabi M. M. Crisco J. J. Vasavada A. Oda T. Cholewicki J. Nibu K. (2001). Mechanical properties of the human cervical spine as shown by three-dimensional load-displacement curves. Spine (Phila Pa 1976) 26 (24), 2692–2700. 10.1097/00007632-200112150-00012 11740357

[B39] Rahyussalim A. J. Aprilya D. Handidwiono R. Whulanza Y. Ramahdita G. Kurniawati T. (2022). The use of 3D polylactic acid scaffolds with hydroxyapatite/alginate composite injection and mesenchymal stem cells as laminoplasty spacers in rabbits. Polym. (Basel) 14 (16), 3292. 10.3390/polym14163292 PMC9416571 36015548

[B40] Silva A. J. C. de Sousa R. J. A. Fernandes F. A. O. Ptak M. Dymek M. Parente M. P. L. (2023). Improvement and validation of a female finite element model of the cervical spine. J. Mech. Behav. Biomed. Mater 142, 105797. 10.1016/j.jmbbm.2023.105797 37058864

[B41] Song M. Zhang Z. Lu M. Zong J. Dong C. Ma K. (2014). Four lateral mass screw fixation techniques in lower cervical spine following laminectomy: a finite element analysis study of stress distribution. Biomed. Eng. Online 13, 115. 10.1186/1475-925x-13-115 25106498 PMC4132205

[B42] Srinivasan S. Kumar S. D. R S. Jebaseelan D. D. Yoganandan N. RajasekaranS (2021). Effect of heterotopic ossification after bryan-cervical disc arthroplasty on adjacent level range of motion: a finite element study. J. Clin. Orthop. Trauma 15, 99–103. 10.1016/j.jcot.2020.10.027 33717922 PMC7920132

[B43] Suk K. S. Kim K. T. Lee J. H. Lee S. H. Lim Y. J. Kim J. S. (2007). Sagittal alignment of the cervical spine after the laminoplasty. Spine (Phila Pa 1976) 32 (23), E656–E660. 10.1097/BRS.0b013e318158c573 17978640

[B44] Sun Z. Lu T. Li J. Liu J. Hu Y. Mi C. (2022). A finite element study on the effects of follower load on the continuous biomechanical responses of subaxial cervical spine. Comput. Biol. Med. 145, 105475. 10.1016/j.compbiomed.2022.105475 35381450

[B45] Takayasu M. Takagi T. Nishizawa T. Osuka K. Nakajima T. Yoshida J. (2002). Bilateral open-door cervical expansive laminoplasty with hydroxyapatite spacers and titanium screws. J. Neurosurg. 96 (1 Suppl. l), 22–28. 10.3171/spi.2002.96.1.0022 11795710

[B46] Tang B. Yang J. Zhang Y. Ren X. Jiang T. Mo Z. (2022). Incorporating strategy in hybrid surgery for continuous two-level cervical spondylosis from a biomechanical perspective. Comput. Methods Programs Biomed. 226, 107193. 10.1016/j.cmpb.2022.107193 36288687

[B47] Tyagi N. Amar Goel S. Alexander M. (2019). Improving quality of life after spinal cord injury in India with telehealth. Spinal Cord. Ser. Cases 5, 70. 10.1038/s41394-019-0212-x PMC6786311 31632728

[B48] Wang H. Q. Mak K. C. Samartzis D. El-Fiky T. Wong Y. W. Luo Z. J. (2011). Spring-back" closure associated with open-door cervical laminoplasty. Spine J. 11 (9), 832–838. 10.1016/j.spinee.2011.07.026 21890423

[B49] Wang J. Wo J. Wen J. Zhang L. Xu W. Wang X. (2022). Laminoplasty versus laminectomy with fusion for treatment of multilevel cervical compressive myelopathy: an updated meta-analysis. Postgrad. Med. J. 98 (1163), 680–688. 10.1136/postgradmedj-2020-139667 37062984

[B50] Wang K. Wang H. Deng Z. Li Z. Zhan H. Niu W. (2017). Cervical traction therapy with and without neck support: a finite element analysis. Musculoskelet. Sci. Pract. 28, 1–9. 10.1016/j.msksp.2017.01.005 28171773

[B51] Wang Y. Liu Y. Zhang A. Han Q. Jiao J. Chen H. (2023). Biomechanical evaluation of a novel individualized zero-profile cage for anterior cervical discectomy and fusion: a finite element analysis. Front. Bioeng. Biotechnol. 11, 1229210. 10.3389/fbioe.2023.1229210 37744254 PMC10512836

[B52] Xiong W. Li F. Guan H. (2015). Tetraplegia after thyroidectomy in a patient with cervical spondylosis: a case report and literature review. Med. Baltim. 94 (6), e524. 10.1097/md.0000000000000524 PMC4602764 25674751

[B53] Xu H. Wu J. Xie H. Wen W. Xu H. Du J. (2022). Biomechanical behaviour of tension-band-reconstruction titanium plate in open-door laminoplasty: a study based on finite element analysis. BMC Musculoskelet. Disord. 23 (1), 851. 10.1186/s12891-022-05804-w 36076212 PMC9454233

[B54] Yuan X. Wei C. Xu W. Gan X. Cao S. Luo J. (2019). Comparison of laminectomy and fusion vs laminoplasty in the treatment of multilevel cervical spondylotic myelopathy: a meta-analysis. Med. Baltim. 98 (13), e14971. 10.1097/md.0000000000014971 PMC6456105 30921202

